# Supplementary material for: The forests of the midwestern United States at Euro-American settlement: Spatial and physical structure based on contemporaneous survey data
Source: PLoS One. 2021 Feb 11;16(2):e0246473. doi: 10.1371/journal.pone.0246473 (PMC7877788; doi:10.1371/journal.pone.0246473)
Supplement: S1 Table — (PDF) [file pone.0246473.s001.pdf]

S4 Table

S4 Table. Modeled density and aboveground biomass in the presettlement and modern periods over 10 regions and 12 ecoregions of the Midwest.

		Presettlement PLS[1]						Modern FIA[2]					
		stem density[3] (trees/ha)			AGB[4] (Mg/ha)			stem density[3] (trees/ha)			AGB[4] (Mg/ha)		
Zone [5]	n [6]	mean	sd	cv (%)	mean	sd	cv (%)	mean	sd	cv (%)	mean	sd	cv (%)
Western Minnesota	2097	29.2	40.8	140	15.1	24.7	164	158	21.6	13.7	110	35.0	31.8
Northeast Minnesota	1506	197.4	65.8	33	64.4	31.3	49	159	18.9	11.9	56	19.4	34.5
Western Upper Peninsula	467	275.0	71.9	27	150.8	32.7	22	220	20.1	9.1	94	24.5	26.1
Northern Wisconsin	1020	243.3	65.7	27	123.3	28.5	23	195	20.1	10.3	89	13.7	15.4
Northern Michigan	809	268.2	74.7	28	125.7	41.6	33	212	24.3	11.5	85	19.2	22.5
Southern Wisconsin	1062	68.3	59.3	87	43.3	40.6	94	172	16.0	9.3	103	14.3	13.9
Western Illinois	2330	27.4	33.6	122	30.1	36.1	120	164	16.0	9.8	145	11.7	8.1
Southern Michigan	1063	205.1	82.2	40	168.9	65.7	39	179	17.7	9.9	113	13.2	11.7
Eastern Indiana	949	245.1	70.3	29	284.6	87.8	31	181	10.9	6.0	147	14.4	9.8
Southern Illinois-Indiana	468	168.1	53.2	32	159.2	46.9	29	190	11.4	6.0	146	8.5	5.8
EPA Ecoregion[7]													
Northern Glaciated Plains	147	0.2	0.4	180	0.1	0.2	163	153	11.2	7.3	117	7.8	6.6
Western Corn Belt Plains	681	8.5	28.3	333	5.9	20.2	343	160	11.1	7.0	138	20.8	15.1
Lake Agassiz Plain	414	14.6	27.8	191	3.0	5.3	178	141	35.0	24.9	70	32.0	45.6
Northern Minnesota Wetlands	355	200.3	96.9	48	36.4	22.7	62	141	18.4	13.0	36	7.1	19.8
Northern Lakes and Forests	2993	237.5	77.2	33	105.9	47.3	45	190	30.1	15.8	76	22.1	29.1
North Central Hardwood Forests	1376	124.5	92.5	74	71.2	55.4	78	182	22.8	12.5	103	19.5	18.9
Driftless Area	630	48.2	51.5	107	34.3	42.5	124	168	6.9	4.1	113	11.9	10.6
Southeastern Wisconsin Till Plains	487	91.8	78.1	85	61.2	45.4	74	168	25.7	15.3	105	14.7	14.1
Central Corn Belt Plains	1199	11.0	18.5	168	12.9	21.8	169	160	18.7	11.7	149	9.9	6.7
Eastern Corn Belt Plains	722	243.7	76.3	31	292.0	95.5	33	178	11.2	6.3	142	14.9	10.4
Southern Michigan/Northern Indiana Drift Plains	834	163.7	72.5	44	149.1	68.4	46	181	16.9	9.3	124	15.9	12.8

Huron/Erie Lake Plains	312	276.5	64.3	23	216.4	52.8	24	173	14.8	8.6	102	13.8	13.6
Interior Plateau	293	229.7	59.7	26	210.3	64.4	31	191	8.9	4.7	151	14.1	9.4
Interior River Valleys and Hills	1323	74.2	64.0	86	76.8	66.8	87	174	13.6	7.8	142	12.0	8.5
Mississippi Alluvial Plain	5	200.0	14.8	7	151.1	20.8	14	178	3.5	1.9	141	1.5	1.1

[1] Values based on smoothed estimates over 8x8 km cells within zone.

[2] Values based on ca. 2010 smoothed estimates over 8x8 km cells within zone.

[3] Mean, standard deviation (sd), and coefficient of variation (cv) of modeled density of stems >20cm dbh across the grid cells.

[4] Mean, standard deviation (sd), and coefficient of variation (cv) of modeled aboveground biomass across the grid cells.

[5] Geographic regions from Fig 5.

[6] Number of 8x8 km cells within zone.
