# Supplementary material for: The forests of the midwestern United States at Euro-American settlement: Spatial and physical structure based on contemporaneous survey data
Source: PLoS One. 2021 Feb 11;16(2):e0246473. doi: 10.1371/journal.pone.0246473 (PMC7877788; doi:10.1371/journal.pone.0246473)
Supplement: S1 Appendix — (PDF) [file pone.0246473.s002.pdf]

S1 Appendix: Data collection and cleaning

We first give an overview of the data cleaning steps applied to all the data, followed by sections specifying the specific data collection and cleaning steps applied to individual spatial regions (mostly at the state level, but with Michigan divided into northern and southern portions).

Overview

Following [1], we excluded line and meander trees (i.e., trees encountered along survey lines as compared to trees located at section or quarter-section corners). Surveyor selection biases for tree size and species appear to have been more strongly expressed for line trees. Meander trees were used to avoid obstacles, such as waterbodies, and so have non-random habitat preferences [2].

We attempted to exclude points in water, including points with information indicating wetlands without trees present; the specifics of how we did this varied by state (described below). Relative to [1], we excluded some additional points in Wisconsin and Minnesota based on information indicating the presence of standing water. Note that points with a single tree might be in areas with low tree density, such that the second tree was too far for the surveyors to mark it. However, in some cases a single tree may be marked because three of the quadrants were inaccessible, generally due to wet conditions. We generally excluded one-tree points if other information suggested this was a result of water rather than because of low tree density, but in many cases it was hard to distinguish between these cases.

Relative to [1] we carried out extensive additional quality control of the northern Michigan data, during which we detected and fixed anomalies in the data (described below). We also excluded a number of additional points with no tree data where the data were judged to be unreliable rather than indicative of low density.

As part of the overall PalEON project, we have been digitizing the PLS data from Illinois, Indiana, and southern Michigan. [1] did not analyze Illinois, Indiana, or southern Michigan, while [3] did analyze these areas, but used an earlier version of the PLS dataset only to estimate composition. The southern Michigan data were digitized from Mylar maps and found to have a variety of errors (described below) that primarily affected stem density, basal area, and biomass rather than composition. For this work, based on the original field notes for southern Michigan, we fixed errors in some points and excluded some points with data judged to be unreliable. For Indiana and Illinois, we now have additional digitized PLS data that were unavailable in [3].

Wisconsin

Copies of the original field notes from Wisconsin are archived at the Wisconsin Board of Commissioners of Public Lands in Madison, Wisconsin. These records have been microfilmed and made available online (http://digicoll.library.wisc.edu/SurveyNotes/SurveyInfo.html or https://glorecords.blm.gov/default.aspx). The Wisconsin point data were digitized by the Mladenoff lab group and have undergone several revisions over the last two decades in an effort to improve data accuracy [2,4-7]. This constitutes the cleaned raw data used in this work and provided in the data product (https://portal.lternet.edu/nis/mapbrowse?scope=msb-paleon&identifier=32&revision=0).

We then processed the cleaned raw data as follows. We excluded 4,088 points with the first tree marked as 'QQ' by the Mladenoff lab as this indicates the presence of water at the point. We also used the vegetation code for each point (documented at the Wisconsin DNR website) to exclude points that might induce a negative bias in our estimates because trees were missing because of standing water. Specifically, we excluded 2,803 points with one tree that were marked as Creek, Marsh, Swamp, Lake, and River. Note that we included points marked as Wet Prairie or “low land, low wet area”, judging these areas to be terrestrial, albeit often wet.

Three areas southwest of Green Bay had no data, because they were Menominee Native American lands and were not surveyed (see Fig 1 in main text). We excluded these points and few other points in Wisconsin (a total of 736 points) for which there was no information on trees at the point. Later surveys on these lands are not included here.

There were 670 points that were missing survey years. The survey year for these was imputed based on nearby points.

Minnesota

Copies of the original submitted field notes from Minnesota are at the Minnesota Historical Society in St. Paul, Minnesota. These records have been digitized by three projects [8,9] and the unpublished Minnesota County Biological Survey of 1996 and are available online at the Minnesota DNR Bearing Tree Database (https://gisdata.mn.gov/dataset/biota-original-pls-bearing-trees). More details are given in [10]. The data used in this work were obtained from the Minnesota DNR by the Mladenoff lab group in earlier work. That version of the data constitutes the cleaned raw data used in this work is provided in this data product: https://portal.lternet.edu/nis/mapbrowse?scope=msb-paleon&identifier=33&revision=0

We then processed the cleaned raw data as follows. We used the vegetation code for each point (documented in [10]) to exclude points that might induce a downward bias in our estimates because trees were missing because of standing water. Specifically, we excluded 20,560 points with no trees or one tree that are marked as Creek, Marsh, Swamp, Lake, and River. We retained 24,243 points with two to four trees in such areas as it is hard to know how much of the area surrounding these corners is under water; this may lead to some downward bias in our density estimates in Minnesota. We excluded 902 points with missing taxon information and missing ecotype as these appeared unusable. Most occurred in far northern Minnesota (the Boundary Waters area) or along straight east-west lines, suggesting problems with the points. We excluded 1,560 Forest, Grove, Bottom and Pine grove points with missing taxa information for all trees, as this is inconsistent with the presence of forest. We included points marked as Wet Prairie, judging these to be terrestrial, albeit often wet.

Northern Michigan

Copies of the original submitted field notes from northern Michigan are at the Michigan State Archives in Lansing, Michigan. These records have been microfilmed and are available online (http://seekingmichigan.org/discover/glo-survey-notes or https://glorecords.blm.gov/default.aspx). Michigan surveyor observations for the Upper Peninsula of Michigan and the northern section of the Lower Peninsula (see Fig 1 of [1]) were digitized by the Mladenoff lab group in earlier work. Co-authors Cogbill and Peters added additional points in Ontonagon, Schoolcraft and Gogebic Counties. The northern Michigan data were further processed to keep one record for locations that had two georeferenced data entries with identical tree information. In cases where there were two georeferenced data entries in the

same location, with either a) one entry providing tree information and the other no tree information or b) both entries having the same tree information except for one attribute (typically the bearing), the point with no information or with less information, respectively, was removed.

The exact point coordinates for Isle Royale appeared to be incorrect (some points are in Lake Superior) and some points appeared to be duplicated. We omitted all data from Isle Royale in the current analysis, but in future work we plan to re-enter the data from the original survey notes.

From initial spot checks in Dickinson County we determined tree diameter and distances were transposed in the data transcription in many cases; these were corrected. Spot checks also indicated that distances in Iosco County (and scattered points in other counties) that had been listed with decimal values needed to be converted from chains to links (multiplying by 100) to standardize with the rest of the database. There were some trees with outlying diameter values greater than 48 inches dbh. All of these were checked carefully in the original field notes and were corrected if necessary.

The cleaned raw data represent the data obtained and processed as described just above and are provided in this data product: https://portal.lternet.edu/nis/mapbrowse?scope=msb-paleon&identifier=31&revision=0.

We then processed the cleaned raw data as follows. Unlike in Minnesota and Wisconsin, we do not have vegetation codes, so we cannot distinguish one-tree points that occur because of water from one-tree points in areas with low density. Some points have qualitative notes but in general these do not indicate the presence of any water. All one-tree points were retained, but they may contribute to a downward bias in density and biomass.

There were 2,810 points with no trees indicated (i.e., with no taxa noted) for which we attempted to determine if these were truly points that had been surveyed that had no nearby trees. All such points (1,316 points) without any surveyor notes were excluded. We included points where the notes indicated no trees (e.g., 'no witness trees', 'no trees convenient', 'no other tree data') but excluded points where the notes indicated water (102 points), lost information (73 points) or that the tree was used as the corner or no other trees were recorded (874 points). In the latter case, we cannot compute a density estimate (it would be infinity) for a point with one tree at zero distance.

Southern Michigan

Copies of the original submitted field notes from southern Michigan are at the Michigan State Archives in Lansing, Michigan. These records have been microfilmed and are available online (http://seekingmichigan.org/discover/glo-survey-notes or https://glorecords.blm.gov/default.aspx). Field notes were transcribed to topographic maps allowing the points to be displayed geographically and were then converted to Mylar maps by Denis Albert and Patrick Comer of the Michigan Natural Features Program for [11]. Ed Schools provided these Mylar maps temporarily to the Williams lab, who digitized them to a point-based ArcGIS shapefile. Co-authors Cogbill and Peters conducted a number of checks described below. When necessary they used the original field notes to check and make corrections.

There were some townships with no survey points (visible as small areas with fewer points per cell, albeit not zero points because the township and grid cell borders are not aligned, in south-central and southeastern Michigan in Fig 1 in main text). Spot checks indicate these are

generally caused by missing data from the original surveys, with the original field notes unavailable for unknown reasons.

We removed points already contained within the northern Michigan dataset. An initial assessment of the data digitized from the mylar maps indicated that at some locations the diameter and distances were transposed during digitization; we corrected these. When the Mylar maps were created, points on the township boundaries were entered twice for approximately 4,000 exterior township corners (mainly on the southern and eastern township borders), resulting in four trees noted per corner when only two were surveyed. We kept the two of the four trees listed when they were located in quadrants inside the township. We excluded 469 points in cases where there was ambiguity because the trees were in quadrants outside the township. We plan to obtain data from these points from the original PLS field notes in future processing. An additional 68 interior township survey points had three or four trees but only two trees were truly surveyed. These were checked against the original PLS field notes and corrected. For entries with azimuths less than zero or greater than 360 we checked the survey notes and corrected as needed. There were some trees with outlying diameter values of greater than 48 inches dbh. All of these were checked carefully in the Mylar maps and/or original field notes and were either corrected or retained only if they were clearly noted in either source.

Due to extensive incomplete data on the Mylar maps, 27 townships in southeastern Michigan (primarily Monroe and Lenawee Counties, with one township in Washtenaw) were re-entered and replaced by the McLachlan lab using the same protocol as used for the Indiana and Illinois data.

The cleaned raw data represent the data obtained and processed as described just above are provided in this data product for southern Michigan excluding the 27 townships noted above: https://portal.lternet.edu/nis/mapbrowse?scope=msb-paleon&identifier=30&revision=0, and in this data product for the 27 townships: https://portal.lternet.edu/nis/mapbrowse?scope=msb-paleon&identifier=29&revision=0.

We then processed the cleaned raw data as follows. The Mylar maps for many areas of southern Michigan (outside of the southeastern Michigan region) have no quarter-section points. The areas with quarter-section points tend to be in savanna / low-density areas. Given this selection bias, we removed all 6,593 quarter-section points that were present. This can be seen in the marked decrease in points per cell in the southern portion of the lower peninsula of Michigan in Fig 1 in main text.

We excluded 40 points with three trees because surveyors in this area were instructed to only mark two trees. These points may be those with a corner tree plus two additional trees but extracting valid data from these points would require looking at the field notes.

Unlike in Minnesota and Wisconsin, we do not have vegetation codes, so we cannot distinguish one-tree points that occur because of water from one-tree points in areas with low density. Some points have qualitative notes but in general these do not indicate the presence of any water. All one-tree points were retained, but they may contribute to a downward bias in density and biomass.

Indiana and Illinois

Copies of the original submitted field notes from Indiana and Illinois are at the Indiana State

Archives in Indianapolis and the Illinois State Archives in Springfield. These records have been microfilmed and are available online in the National Archives (https://catalog.archives.gov/id/566714). Data from Indiana and Illinois were purchased from the Indiana State Archives (Commission on Public Records, Indiana State Archives, Indianapolis) Indiana) and Hubtack Document Resources (https://new.hubtack.com/), respectively, and processed by the McLachlan lab. Data entry for these states is ongoing. Originally, townships to digitize were chosen to provide an even distribution across both states. Since then, specific areas of each state (e.g., the Kankakee watershed, the Yellow River watershed, the savanna-closed forest transition north to south in Illinois and west to east in Indiana, and locations of US Forest Service Forest Inventory Analysis (FIA) plots) have been chosen to complement ongoing projects in the lab. PLS land notes are transcribed by undergraduates in the lab. These data are then subjected to an initial QA/QC check by the original readers, followed up by a second QA/QC check by a different individual in the lab, georeferenced to the section and quarter-section locations, and finally reviewed for a final set of QA/QC checks. The R code used for the QA/QC checks and georeferencing are available in a GitHub repository at: https://github.com/PalEON-Project/IN_ILTownshipChecker.

The cleaned raw data represent the data obtained and processed as described just above and are provided in this data product for Indiana : https://portal.lternet.edu/nis/mapbrowse?scope=msb-paleon&identifier=27&revision=0, and this data product for Illinois: https://portal.lternet.edu/nis/mapbrowse?scope=msb-paleon&identifier=28&revision=0.

We then processed the cleaned raw data as follows. There were 18 points in the Illinois data near the Illinois-Wisconsin border that were very close to points in Wisconsin. These were removed to avoid near-duplication of points. A small number of points were missing survey years. The survey year for these was imputed based on nearby points.

Points recorded as Water (where surveyor notes indicated standing water) or having no data (which is not the same as having no trees) were omitted. Points recorded as Wet (where surveyor notes indicated water was ephemeral) were included as these were judged to be terrestrial. All one-tree survey points are retained as the survey information (including qualitative notes by the surveyors) indicates that such points were because of low density and not the presence of water.

Other notes

We excluded 127 points in Minnesota, Wisconsin, and northern Michigan in which at least one tree was marked as dead (or 'dry') or where the taxon was judged 'indeterminable' by the Mladenoff lab. In almost all these cases, there would be fewer than two live trees after excluding the dead or unknown cases.

Exclusions and adjustments for density calculation

We used bearing angle information to screen and correct for points where surveyors may not have followed the PLS instructions. Specifically, we searched for and found 9,602 four-tree points where the two nearest trees fell in the same quadrant. We excluded these points as this indicates the surveyors did not follow the survey instructions, and there is no rigorous way to use the Morisita estimator to estimate density for these points. In cases where information on the quadrant was missing for one or both trees of the two closest trees, we assumed they fell in different quadrants and did calculate stem density.

We removed 3,629 points with one tree at a distance of zero or missing distance as it is unclear what density to estimate for such points. Many of these corners have a single corner tree with a zero distance (presumably a “corner tree” used as the corner post), which our density estimator would assign a density of infinity. We removed 1,821 points with two trees and either distance missing. We also removed 131 points with two trees at distance zero. Points with one of two trees at distance zero do allow estimation of density using the Morisita estimator and were included.

We estimated density for one-tree points as 0.3146 stems per hectare. This density estimate is equal to one tree in a circle of radius equal to 500 links (approximately 100 m), which approximates how far a surveyor might have gone to find a second tree. Surveyors were instructed to find two trees when possible, so the presence of only one tree generally indicates low density. While 500 links is arbitrary, our results should be insensitive to the exact value of the near-zero density that we use in such cases.

We truncated estimated densities at 10,000 stems per hectare (one tree per square meter) to reduce the influence of high-density outliers, which resulted in truncating 139 points when estimating stem density itself and 246 points when estimating stem density for the biomass and basal area estimation (i.e., omitting the scaling to trees greater than or equal to 8 inches dbh as discussed below).

References

[1] Goring SJ, Williams JW, Mladenoff DJ, Cogbill CV, Record S, Paciorek CJ, et al. Novel and lost forests in the upper Midwestern United States, from new estimates of settlement-era composition, stem density, and biomass. PLoS One. 2016;11: e0151935.

[2] Liu F, Mladenoff DJ, Keuler NS, Moore LS. Broadscale variability in tree data of the historical Public Land Survey and its consequences for ecological studies. Ecological Monographs. 2011;81: 259-275.

[3] Paciorek CJ, Goring SJ, Thurman AL, Cogbill CV, Williams JW, Mladenoff DJ, et al. Statistically-estimated tree composition for the northeastern United States at Euro-American settlement. PloS One. 2016;11: e0150087.

[4] Radeloff VC, Mladenoff DJ, Boyce MS. A historical perspective and future outlook on landscape scale restoration in the northwest Wisconsin pine barrens. Restoration Ecology. 2000;8: 119–126.

[5] Manies KL, Mladenoff DJ. Testing methods to produce landscape-scale presettlement vegetation maps from the US Public Land Survey records. Landscape Ecology. 2000;15: 741–754.

[6] Mladenoff DJ, Dahir SE, Nordheim EV, Schulte LA, Guntenspergen GG. Narrowing historical uncertainty: Probabilistic classification of ambiguously identified tree species in historical forest survey data. Ecosystems. 2002;5: 539–553.

[7] Schulte LA, Mladenoff DJ, Nordheim EV. Quantitative classification of a historic northern Wisconsin (USA) landscape: Mapping forests at regional scales. Canadian Journal of Forest Research. 2002;32: 1616–1638.

[8] Grimm EC. An ecological and paleoecological study of the vegetation in the Big Woods region of Minnesota. Ph.D. dissertation, University of Minnesota; 1981.

[9] Almendinger JC. The late-Holocene development of jack pine forests on outwash plains, north-central Minnesota. Ph.D. dissertation, University of Minnesota. 1985.

[10] Almendinger JC. Minnesota's bearing tree database. Biological Report No. 56. Minnesota Department of Natural Resources; 1997.

[11] Albert DA, Comer PJ, Enander H. Atlas of early Michigan's forests, grasslands, and wetlands. Lansing, Michigan: Michigan State University Press; 2008.
