# Supplementary material for: The forests of the midwestern United States at Euro-American settlement: Spatial and physical structure based on contemporaneous survey data
Source: PLoS One. 2021 Feb 11;16(2):e0246473. doi: 10.1371/journal.pone.0246473 (PMC7877788; doi:10.1371/journal.pone.0246473)
Supplement: S2 Appendix — (PDF) [file pone.0246473.s003.pdf]

S2 Appendix: The Morisita plotless density estimator and correction factors

Deputy surveyors were instructed (ca. 1805) to mark “two or more adjacent trees in opposite direction as nearly as may be” at each section or quarter-section corner [1]. The procedure was implemented from the earliest PLS surveys in Illinois as evident by two witness trees invariably being recorded. Later the survey instructions required that witness trees be in different sections; this two-tree procedure remained unchanged on exterior and interior lines through the 1850s in Ohio, Illinois, Indiana, and Michigan. In 1846 the instructions for Wisconsin and Iowa (including Minnesota) changed to include four witness trees at township and section corners and two trees in opposite sections at quarter-section corners [1].

As a result, there are three common sampling designs in the Midwest PLS data. The predominant pattern was for two-tree corners (85%). Exterior corners in early surveys in the southern part of the domain were sampled with two trees on the same side inside the township. At the remaining exterior and all interior corners in all Midwestern states, the sampling design generally involved two trees in opposite halves or dominant “opposite” with a mixture from “adjacent” quadrants. Four-tree corners (9% of all Midwest) were prevalent in the northern part of the domain, generally surveyed after 1846. Virtually all these four-tree samples (95%) had one witness tree in each quadrant/section and the majority (61%) were at interior section corners.

Given this heterogeneity, we corrected the Morisita estimator to minimize error due to different sampling geometries and several known surveyor biases [2-9], as discussed in [10]. Our approach allows for spatial variation in surveyor methods by applying correction factors based on the empirical sample geometry and known surveyor biases deviating from this design. These estimates are based on empirical examination of the underlying data and have been validated using simulations on stem-mapped stands. The correction factors vary by state (and in some cases regions within a state), date, internal versus external point, section versus quarter-section, and two- versus four-tree points and can be found in our GitHub repository. There are four correction factors: *kappa* accounts for the sampling design (two- versus four-tree points and where the two trees are located relative to quadrants and halves), *theta* for sector bias, *zeta* for azimuthal censoring, and *phi* for inclusion of trees less than 8 inches dbh, as discussed in [10].

References

[1] White CA. A history of the rectangular survey system. US Department of the Interior, Bureau of Land Management; 1983.

[2] Liu F, Mladenoff DJ, Keuler NS, Moore LS. Broadscale variability in tree data of the historical Public Land Survey and its consequences for ecological studies. Ecological Monographs. 2011;81: 259-275.

[3] Bouldin J. Some problems and solutions in density estimation from bearing tree data: a review and synthesis. Journal of Biogeography. 2008;35: 2000–2011.

[4] Manies KL, Mladenoff DJ, Nordheim EV. Assessing large-scale surveyor variability in the historic forest data of the original US Public Land Survey. Canadian Journal of Forest Research. 2001;31: 1719–1730.

[5] Kronenfeld BJ, Wang YC. Accounting for surveyor inconsistency and bias in estimation of tree density from presettlement land survey records. Canadian Journal of Forest Research. 2007;37: 2365–2379.

[6] Williams MA, Baker WL. Testing the accuracy of new methods for reconstructing historical structure of forest landscapes using GLO survey data. Ecological Monographs. 2011;81: 63–88.

[7] Hanberry BB, Fraver S, He HS, Yang J, Dey DC, Palik BJ. Spatial pattern corrections and sample sizes for forest density estimates of historical tree surveys. Landscape Ecology. 2011;26: 59–68.

[8] Hanberry BB, Palik BJ, He HS. Comparison of historical and current forest surveys for detection of homogenization and mesophication of Minnesota forests. Landscape Ecology. 2012a;27: 1495–1512.

[9] Hanberry BB, Yang J, Kabrick JM, He HS. Adjusting forest density estimates for surveyor bias in historical tree surveys. The American Midland Naturalist. 2012b;167: 285–306.

[10] Goring SJ, Williams JW, Mladenoff DJ, Cogbill CV, Record S, Paciorek CJ, et al. Novel and lost forests in the upper Midwestern United States, from new estimates of settlement-era composition, stem density, and biomass. PLoS One. 2016;11: e0151935.
