# Supplementary material for: The forests of the midwestern United States at Euro-American settlement: Spatial and physical structure based on contemporaneous survey data
Source: PLoS One. 2021 Feb 11;16(2):e0246473. doi: 10.1371/journal.pone.0246473 (PMC7877788; doi:10.1371/journal.pone.0246473)
Supplement: S3 Appendix — (PDF) [file pone.0246473.s004.pdf]

S3 Appendix: Model selection using cross-validation

The absolute error loss for the biomass models fitted on the log scale was lower than for models fitted on the original scale (compare S3 Tables 1 and 2). With regard to coverage, the models fitted on the original scale without log transformation had a poor tradeoff of coverage and interval lengths (not shown). For example, for our final choices of k=2500 for occupancy and k=3500 for potential, for a 90% uncertainty interval, the coverage was 94.8% with a median interval length of 188 for the potential model on the original scale, compared to coverage of 85.5% with a median interval length of 97 for the potential model on the log scale. While the 85.5% coverage is less than the desired coverage of 90%, we judge that the modest undercoverage is acceptable in light of the much shorter interval lengths. In addition, the uncertainty was roughly constant regardless of the value of the point estimate when working on the original scale, while the models using the log scale had uncertainty that increased with the size of the point estimate. This scaling of variance with mean (similar to that in a Poisson distribution) when using the log scale makes intuitive sense given the lower bound of zero.

S3 Table 1: Weighted absolute error for total biomass per cell (Mg/ha) by number of basis functions for occupancy model (rows) and potential biomass model (columns) with potential model fit on original scale. Cells are weighted based on the number of PLS points in the cell.

	k for potential model								
k for occu-pancy model	100	250	500	1000	1500	2000	2500	3000	3500
100	38.47	36.35	34.91	33.81	33.43	33.34	32.58	32.54	32.51
250	38.05	36.02	34.60	33.51	33.14	33.05	32.30	32.26	32.23
500	37.75	35.67	34.29	33.23	32.87	32.78	32.04	31.99	31.97
1000	37.46	35.37	33.99	32.97	32.62	32.52	31.80	31.76	31.74
1500	37.35	35.24	33.86	32.85	32.49	32.40	31.67	31.63	31.61
2000	37.35	35.24	33.86	32.84	32.48	32.39	31.67	31.62	31.60
2500	37.17	35.07	33.68	32.66	32.31	32.22	31.50	31.45	31.43
3000	37.15	35.05	33.65	32.62	32.27	32.18	31.45	31.41	31.39
3500	37.15	35.05	33.65	32.62	32.27	32.17	31.45	31.41	31.39

S3 Table 2: Weighted absolute error for total biomass per cell (Mg/ha) by number of basis functions for occupancy model (rows) and potential biomass model (columns) with potential model fit on log scale. Cells are weighted based on the number of PLS points in the cell.

	k for potential model								
k for occu-pancy model	100	250	500	1000	1500	2000	2500	3000	3500
100	37.45	35.27	33.41	32.00	31.41	31.22	30.43	30.32	30.25
250	37.10	34.98	33.19	31.81	31.23	31.05	30.26	30.16	30.09
500	36.78	34.65	32.90	31.57	31.01	30.83	30.05	29.95	29.88
1000	36.52	34.38	32.65	31.38	30.83	30.65	29.88	29.78	29.71
1500	36.40	34.26	32.53	31.26	30.70	30.53	29.77	29.67	29.61
2000	36.39	34.26	32.50	31.23	30.68	30.51	29.75	29.65	29.58
2500	36.26	34.10	32.36	31.10	30.56	30.39	29.64	29.54	29.48
3000	36.24	34.08	32.33	31.08	30.53	30.36	29.62	29.52	29.46
3500	36.23	34.08	32.33	31.07	30.52	30.35	29.60	29.51	29.44

Cross-validation results for total stem density were qualitatively similar to those for biomass with regard to how values vary with the number of basis functions (not shown). With regard to comparing results on the original and log scales, for k=2500 for occupancy and k=3500 for potential, the weighted absolute error was 41.5 and 40.1 stems/ha per cell for the original and log scales, respectively. Coverage was 91.5% and 85.4%, respectively, and the median interval lengths were 205 and 185, respectively.

Cross-validation results for taxon-level estimates are harder to interpret because each taxon has its own results. Also, for grid cells outside the range limit of a taxon, estimates and intervals are generally very close to zero. As a result, it is difficult to know how best to aggregate across taxa for summarization. Nevertheless, the variation in cross-validation results with respect to the number of basis functions (not shown) was qualitatively similar to the results for total biomass. For k=2500 for occupancy and k=3500 for potential, the average coverage (across taxa) of 90% uncertainty intervals was 97.8% for the original scale and 93.6% for the log scale, with a mean (across taxa) of median interval lengths (across cells) of 11.1 and 3.9 for the original and log scales, respectively.

Based on the cross-validation results we chose to fit models on the log scale. We also chose k=2500 for the occupancy models (for stem density, basal area, and biomass, and for total and taxon-level fitting) and k=3500 for the potential models. While values of k>2500 for occupancy reduced the estimated absolute error loss (i.e., improved the fits) slightly (see S3 Tables 1 and 2), larger k values increased computational time, so we chose to use k=2500. We did not assess k>3500. Based on the diminishing reductions in the loss as k increases beyond 2000 or 2500, it is unlikely that larger values of k would produce substantively important improvements in prediction.
