# Supplementary material for: The forests of the midwestern United States at Euro-American settlement: Spatial and physical structure based on contemporaneous survey data
Source: PLoS One. 2021 Feb 11;16(2):e0246473. doi: 10.1371/journal.pone.0246473 (PMC7877788; doi:10.1371/journal.pone.0246473)
Supplement: Attachment — Submitted filename: Response_to_Reviewers.pdf [file pone.0246473.s005.pdf]

Dear Dr. Zang,

We are submitting a revised version of PONE-D-20-31336. We thank you and the reviewers for thoughtful comments and suggestions. We've responded to the comments point by point below.

Note that in our responses, the line numbers refer to the tracked changes version of the resubmitted manuscript.

Editor comments

1. *PLOS style requirements*:
   Response: We've revised our file names to try to follow the requirements. In general, we believe we have satisfied the style requirements. Please let us know of anything we have overlooked.

2. *We note that Figures 1-5 in your submission contain map images which may be copyrighted.*
   Response: We're not sure why you think the figures may be copyrighted. We created these figures using the data presented in this work. The copyright is held by us (the authors), so we shouldn't need to seek permission from anyone.

3. *Please include captions for your Supporting Information files at the end of your manuscript, and update any in-text citations to match accordingly.*
   Response: All of the tables in our Supporting Information files have captions (we have two tables in S3 Appendix, an S4 Table, and no supporting figures). So we're not sure what this request is referring to. We're happy to make modifications if given additional guidance.

Reviewer #1 comments

- *In my opinion, the "Methods" part is too long and has many detailed information and description, it may be better to present the Methods in a concise way and move the detailed description into a supplementary material. In addition, there are a few points the authors need to clarify in their manuscript.*
  Response: We've moved a number of additional details about the data cleaning/preprocessing (lines 159-187 and 210-237 in the original submission) to the supplementary material (lines 9-41, 237-265 in S1 Appendix). If required, we could move more details of the statistical smoothing as well. However, the smoothing approach we have taken is a new statistical approach to such forest structure data and thus represents a core part of what is new about this work compared to [7], so we feel it appropriate to leave this in the main methods section.

- *1. Line 51, give a concise description of the "Historical datasets", what kinds of data are included in the "Historical datasets".*
  Response: We've changed "datasets" to be "vegetation surveys" in line 51 of the resubmission to be more precise. Combined with the extensive discussion of the survey data in lines 65-76 of the resubmission, we feel that the description of the data used is clear.

- *2. Line 57, from line 53 to 57, the authors listed several aspects in which the datasets are potentially useful and could be applied to study those questions. I suggest the authors illustrate the applications of their dataset to those questions using proper examples.*
  Response: We've added a number of citations in lines 54-57 of the resubmission that illustrate such uses of these datasets.

- *3. Line 87, briefly describe the results obtained by [7], and explain the relationship between this work and [7], and highlight the novelty and significance of the current work.*
  Response: We've added more detail on what was done in [7] in lines 87-91 of the resubmission. We've retained previous text (lines 91-95 in the resubmission) that also discussed how these results differ from [7]. Finally, we added text (lines 95-96 of the resubmission) to indicate that in this work we focus on analyzing the biogeographic patterns at the time of settlement (i.e., with less focus on comparing to the modern vegetation than in [7]).

- *4. Line 101, from line 92 to 101, the authors summarized the main idea of their manuscript. I suggest the authors clearly present the significance of their work at the end of the "Introduction".*
  Response: We've added text in lines 109-115 of the resubmission to this effect.

Reviewer #2 comments

- *Somehow the formatting of Table 2 did not allow its complete printing, but I did not find any problem with the contents.*
  Response: Yes, we are a bit confused about how to present these wide tables. We tried to follow the PLOS requirements but welcome further guidance.

- *I would like to see larger fonts in the legends of Fig. 2.*
  Response: We have increased the font size.

- *Also, I am not sure if you really need lines 92 to 101. It is unusual to provide these mini introductions to the other sections of the manuscripts, but it could be the writing style of the authors and I will leave it to them.*
  Response: the first author is a statistician, and it is common in statistics papers to present an overview of the remainder of the paper at the end of the introduction. We'd like to leave this material, but if the Editor requests that we remove it, we will do so.

Other changes

In revising the manuscript, we have also made some other minor wording changes in various places in the document. These can of course be seen in the tracked changes version of the main manuscript and the S1 Appendix and S2 Appendix.
